# HIV risk behaviors of male injecting drug users and associated non-condom use with regular female sexual partners in north-east India

**DOI:** 10.1186/1477-7517-11-5

**Published:** 2014-02-13

**Authors:** Ritu Kumar Mishra, Deepika Ganju, Sowmya Ramesh, Melody Lalmuanpuii, Langkham Biangtung, Chumben Humtsoe, Niranjan Saggurti

**Affiliations:** 1 Project ORCHID, Emmanuel Hospital Association, Mission Compound, Panbazar, Guwahati, Assam 781001, India; 2 HIV and AIDS Program, Population Council, New Delhi 110003, India

**Keywords:** Injecting drug users, Inconsistent condom use, Regular sexual partners, HIV risk, High-risk behaviors

## Abstract

**Background:**

In the context of increasing HIV prevalence among women in regular sexual partnerships, this paper examines the relationship between male injecting drug users' (IDUs) risky injecting practices and sexual risk behaviors with casual partners and inconsistent condom use with regular partners.

**Methods:**

Data were drawn from the behavioral tracking survey, conducted in 2009 with 1,712 male IDUs in two districts each of Manipur and Nagaland states, in north-east India. IDUs' risky behaviors were determined using two measures: ever shared needles/syringes and engaged in unprotected sex with casual paid/unpaid female partners in the past 12 months. Inconsistent condom use with regular sexual partners (wife/girlfriend) in the past 12 months was assessed in terms of non-condom use in any sexual encounter.

**Results:**

More than one-quarter of IDUs had shared needles/syringes, and 40% had a casual sexual partner. Among those who had casual sexual partners, 65% reported inconsistent condom use with such partners. IDUs who shared needles/syringes were more likely to engage in unprotected sex with their regular partners (95% vs 87%; adjusted OR = 2.31, 95% CI = 1.30–4.09). Similarly, IDUs who reported inconsistent condom use with casual partners were more likely to report unprotected sex with their regular partners (97% vs 66%; adjusted OR = 18.14, 95% CI = 6.82–48.21).

**Conclusion:**

IDUs who engage in risky injecting and/or sexual behaviors with casual partners also report non-condom use with their regular sex partners, suggesting the high likelihood of HIV transmission from IDUs to their regular sexual partners. Risk reduction programs for IDUs need to include communication about condom use in all relationships in an effort to achieve the goal of zero new infections.

## Background

Recent studies have shown that the face of the HIV epidemic in India is changing; while new infections in the adult population have reduced dramatically, HIV infection rates among women have increased over the past decades, mainly as a result of male-to-female transmission within marriage and intimate partnerships [1-4]. According to current estimates, women account for 39% of India's HIV-positive population [3], of whom the majority have acquired HIV due to their husband's or regular sexual partner's high-risk behaviors [4-7]. Reflecting these findings, a study in south India indicates that most new HIV infections (80%–85%) among women originated from their husbands/main partners, who acquired HIV from female sex workers [8].

The regular sexual partners of high-risk males, such as migrants and injecting drug users (IDUs), are at elevated risk for HIV [9,10]. Evidence has shown, for example, that women with a migrant husband are more than two times more likely to be HIV-positive than those with a non-migrant husband [7], and monogamous non-injecting spouses of IDUs are ten times more likely to be HIV-positive than general population women [11]. As documented in several studies, IDUs' dual risk behaviors—sharing needles/syringes and unprotected sex—make their sexual partners much more vulnerable to acquiring HIV [11-13]. High HIV prevalence (7.14%) among IDUs [3] and continued needle/syringe sharing [14,15] not only increase the risk of HIV acquisition but also increase the transmission of HIV to their injecting partners. Further, a large proportion of IDUs are sexually active [13-15]. In Manipur, 10% of married IDUs engage in sex with female sex workers and 55% engage in sex with regular female partners outside marriage [12]. Notably, condom use is limited in sex with casual paid/unpaid female partners [11-19]. While HIV transmission among IDUs through blood contact is considerably higher than through unprotected sex [20], IDUs' risky sexual practices, including low condom use, creates an environment for the sexual transmission of HIV [10].

Studies from India have shown that the female partners of IDUs who share needles/syringes are at high risk for HIV; as reported in a study in Chennai, 5% of the wives of HIV-positive IDUs who reported risky injection practices were infected with the virus [19]. Other studies have shown that the female partners of IDUs who engage in sex with casual/regular partners are at risk for HIV; in Manipur, 45% of the wives of HIV-infected IDUs who reported risky sexual behaviors were found to be HIV-positive [18].

In the north-eastern states of India, the HIV epidemic is driven by injecting drug use—HIV prevalence among IDUs in 2010–2011 in Manipur was 12.89% and 2.21% in Nagaland; adult HIV prevalence in 2009 was 1.40% in Manipur and 0.78% in Nagaland [21].

While there is evidence of the close link between IDUs' risky injecting and sexual practices outside marriage, limited information is available on the association between male IDUs' risk behaviors, that is, unsafe injecting drug use and unprotected sex with casual paid/unpaid female partners, and inconsistent condom use with regular sexual partners (defined as wives or girlfriends) in high HIV prevalence settings in India. To address this knowledge gap, this paper examines the relationship between IDUs' HIV risk behaviors—unsafe injection practices and unprotected sex with casual paid/unpaid partners—and inconsistent condom use with regular sexual partners.

## Methods

### Study design

This study is based on data from the behavioral tracking survey (BTS), a cross-sectional survey that collected information on HIV risk behaviors, HIV knowledge and exposure to harm reduction interventions. The survey was conducted in 2009 among IDUs in two districts of Manipur (Ukhrul and Chandel) and two districts of Nagaland (Kiphire and Zunheboto). Written consent was obtained for the study from all participants. The study was reviewed and approved by the institutional review board (IRB) of the Emmanuel Hospital Association Ethics Committee, New Delhi.

### Sampling

Participants for the BTS were recruited using respondent-driven sampling (RDS). RDS is a validated probability sampling method based on conventional snowball sampling used to recruit hidden populations such as sex workers and IDUs [22]. This method has been used in several HIV biological and behavioral surveillance studies [23]. In RDS, respondents are recruited through an initial pool of accessible contacts (seeds), who are the starting point for a system of recruitment based on coupons and financial incentives.

For this study, the initial four seeds in each district were purposively selected to reflect the diversity of demographic characteristics. Three coupons were provided to each seed participant for distribution among IDUs in their network. Further, each subsequent participant was provided three coupons to recruit three additional IDUs to participate in the study, and this process was continued until the desired sample size was achieved. The sample composition reached equilibrium within eight to ten recruitment waves. Only people who presented coupons were eligible to participate in the study. A detailed description of the sampling design has been published elsewhere [14].

A sample size of 400 was estimated for this study, based on the ability to detect a change in proportions of 12 percentage points from estimated baseline values of 50%, with power of 80% and an alpha error of 5% for a two-sided test. A design effect of 1.5 was applied to account for intra-class correlation. In all, 1,712 respondents, 836 from Manipur and 876 from Nagaland, were recruited for this study.

### Data collection

To be eligible for participation in the interview, individuals had to be male, 18 years of age or older, and have injected drugs for non-medical reasons at least once in the 6 months prior to the survey. Screening of participants for eligibility was done by peer educators who were acquainted with the local context of injecting drug use. A standardized questionnaire, adapted from one previously used in a large-scale behavioral survey with IDUs in India [24], was used in the present study. The questionnaire collected information on sociodemographic characteristics, drug use and injecting practices, sexual behavior, and condom use.

### Measures

The main dependent measures considered in this study were as follows: (a) had a regular female sexual partner in the 12 months prior to the survey and (b) inconsistent condom use with a regular female partner. Participants were defined as inconsistent condom users if they had not used a condom at every sexual encounter with a regular female partner.

Two key independent variables were considered in this study: (1) needle/syringe-sharing practices ever and (2) risky sexual behaviors with casual paid/unpaid sexual partner in the past 12 months. Information regarding needle/syringe sharing was recorded via a single item in the questionnaire and was defined as having injected with needles/syringes previously used by someone else. For assessing the risky sexual behaviors, the survey questionnaire included the following questions: (a) Have you had sex with non-spousal, casual (paid/unpaid) sexual partners in the past 12 months? (b) How frequently did you use condoms in sex with such partners? Based on responses to these two questions, respondents were grouped into three mutually exclusive categories: (a) consistent condom users (defined as those who used condoms at every sexual encounter with casual paid/unpaid sexual partners); (b) respondents who did not have a casual paid/unpaid sexual partner, and (c) inconsistent condom users (defined as those who did not use condoms consistently at every sexual encounter with casual paid/unpaid sexual partners).

Sociodemographic characteristics included age, literacy, marital status, and employment status. Age was measured as a continuous variable and grouped into three categories: 18–24, 25–29, and 30 years or older. Literacy was defined as the ability to both read and write. Marital status was categorized as the respondent being never married or ever married, and employment status was recorded as a response to direct questions and grouped into six categories: unemployed, student, self/salaried employment, laborer (agricultural/non-agricultural), small-time business/trade, and others (including other occupations such as unskilled labor and selling drugs).

Age at drug initiation was recorded as a continuous variable and grouped into four categories: less than 18, 18–24, 25–29, and 30 years and older. Drug use was dichotomized as frequent (defined as injecting at least four times in the past week) and less frequent (defined as injected less than four times in the past week).

### Statistical analysis

The study used the RDS method to recruit participants. In most cases, RDS data are analyzed using RDSAT software, which generates weighted estimates of proportions [22]. However, RDSAT software cannot calculate bivariate or multivariate statistics; therefore, in this study, STATA (Version 11.0) was used for all analyses. Descriptive analyses were undertaken for all sociodemographic variables and sexual and injecting behaviors. Bivariate analyses explored the relationship between IDUs' risky injecting practices and sexual practices with casual partners against key sociodemographic characteristics. Further, a multivariate logistic regression analysis was conducted to estimate the strength of association between IDUs' risk behaviors and inconsistent condom use with regular sexual partners, after adjusting for age, marital status, literacy, occupation, age at first injection, frequency of injecting drugs, and state.

## Results

In both Manipur and Nagaland, most respondents were less than 30 years of age, literate, and had never married (Table 1). Around two fifths were unemployed. The frequency of drug use differed across the states: while 80% of respondents in Manipur frequently injected drugs (at least four times in the last week), only 36% in Nagaland were frequent drug injectors. Around one quarter or more IDUs in both states had shared needles/syringes. Although a higher proportion of IDUs in Nagaland (46%) than in Manipur (35%) had a casual paid/unpaid sexual partner in the past 12 months, unprotected sex with these partners was higher in Manipur (72%) than in Nagaland (60%).

**Table 1:** Sociodemographic characteristics, injecting practices, and sexual risk behaviors of injecting drug users

**Characteristics**	**Manipur *N*** **= 836**	**Nagaland *N*** **= 876**	**Total *N*** **= 1,712**
	**% ( *n *)**	**% ( *n *)**	**% ( *n *)**
Age			
18–24	28.7 (240)	48.4 (424)	38.8 (664)
25–29	25.2 (211)	30.4 (266)	27.9 (477)
30+	46.1 (385)	21.2 (186)	33.4 (571)
Literacy			
Illiterate	10.3 (86)	26.6 (233)	18.6 (319)
Literate	89.7 (750)	73.4 (643)	81.4 (1,393)
Marital status			
Never married	58.0 (485)	68.2 (597)	63.2 (1,082)
Ever married	42.0 (351)	31.8 (279)	36.8 (630)
Employment status			
Unemployed	32.5 (272)	45.2 (396)	39.0 (668)
Student	6.6 (55)	13.1 (115)	9.9 (170)
Self/salaried employment	20.7 (173)	15.0 (131)	17.8 (304)
Laborer (agricultural/non-agricultural)	32.9 (275)	22.0 (193)	27.3 (468)
Small business/trade	5.5 (46)	2.7 (24)	4.1 (70)
Others^a^	1.8 (15)	1.9 (17)	1.9 (32)
Age at first injection (years)			
< 18	7.5 (63)	5.0 (44)	6.3 (107)
18–24	57.8 (483)	71.9 (630)	65.0 (1,113)
25–29	20.1 (168)	19.5 (171)	19.8 (339)
30+	14.6 (122)	3.5 (31)	8.9 (153)
Frequency of injecting drugs^b^			
Less frequent	20.0 (167)	64.4 (564)	42.7 (731)
Frequent	80.0 (669)	35.6 (312)	57.3 (981)
Shared needles/syringes			
No	67.1 (561)	74.7 (654)	71.1 (1,215)
Yes	32.8 (274)	25.3 (221)	29.0 (495)
Had a casual paid/unpaid sexual partner^c^			
No	65.2 (545)	54.5 (477)	59.7 (1,022)
Yes	34.8 (291)	45.6 (399)	40.3 (690)
Condom use with casual paid/unpaid sexual partner^c, d, e^			
Consistent	28.5 (83)	40.6 (162)	35.5 (245)
Inconsistent	71.5 (208)	59.4 (237)	64.5 (445)
Had a regular sexual partner^f^			
No	46.9 (392)	30.1 (264)	38.3 (656)
Yes	53.1 (444)	69.9 (612)	61.7 (1,056)
Condom use with regular sexual partner^d, f, g^			
Consistent	11.5 (51)	10.3 (63)	10.8 (114)
Inconsistent	88.5 (393)	89.7 (548)	89.2 (941)

^a^Include other occupations such as unskilled labor and selling drugs. ^b^Frequent includes those who injected at least four times in past week; less frequent includes those who injected less than four times in the past week. ^c^Casual paid/unpaid sexual partner: women with whom IDUs had sex in the last 12 months in exchange for cash/drugs/kind and women other than main steady partner/spouse with whom IDUs had sex but did not pay. ^d^Consistent: used condoms in every sexual encounter; inconsistent: did not use condoms consistently in every sexual encounter. ^e^Among those who had a casual paid/unpaid sexual partner. ^f^Regular sexual partner: main/steady sexual partner (spouse or girlfriend) in the past 12 months. ^g^Among those who had a regular sexual partner. Sum of the categories may not total to 100 due to missing responses.

Over one fourth (29%) of participating IDUs reported sharing needles/syringes, and two fifths (40%) reported engaging in sex with casual female partners (Table 2). Almost two thirds of those who had sex with casual female partners reported inconsistent condom use. There was an overlap among IDUs who shared needles and engaged in sex with casual female partners: of those who shared needles, 40% also engaged in sex with a casual female partner, and of those reporting sex with casual female partners, 29% also shared injections/syringes. As seen in Table 2, the significant descriptors of unsafe injecting practices were age, education, marital status, occupation, age at first injecting drug use, and frequency of injecting use (*p* < 0.05). Similar findings were noted for inconsistent condom use in sex with casual female partners (*p* < 0.001), except for frequency of injecting drugs (*p* = 0.059).

**Table 2:** Association between IDUs' background characteristics, needle/syringe sharing and condom use with casual partners

**Characteristics**	**Shared needles/syringes**			**Condom use with casual paid/unpaid sexual partner**^**a**^			
	**No**	**Yes**	***P *** **value**	**Consistent condom use**	**No casual paid/unpaid sexual partner**	**Inconsistent condom use**^**a**^	***p *** **value**
	**% ( *n *)**	**% ( *n *)**					
				**% ( *n *)**	**% ( *n *)**	**% ( *n *)**	
Condom use with casual paid/unpaid sexual partner^a^			0.042				
Consistent condom use	15.6 (189)	11.3 (56)		–	–	–	
No casual paid/unpaid sexual partner	59.5 (723)	60.0 (297)		–	–	–	
Inconsistent condom use^b^	24.9 (303)	28.7 (142)		–	–	–	
Shared needles/syringes							0.042
No	–	–		77.1 (189)	70.9 (723)	68.1 (303)	
Yes	–	–		22.9 (56)	22.1 (297)	31.9 (142)	
Age (years)			<0.001				<0.001
18–24	42.9 (521)	28.7 (142)		60.4 (148)	30.6 (313)	45.6 (203)	
25–29	27.1 (329)	29.9 (148)		26.9 (66)	25.2 (258)	34.4 (153)	
30+	30.0 (365)	41.4 (205)		12.7 (31)	44.1 (451)	20.0 (89)	
Literacy			<0.014				<0.001
Illiterate	17.1 (208)	22.2 (110)		9.0 (22)	21.3 (218)	17.8 (79)	
Literate	82.9 (1007)	77.8 (385)		91.0 (223)	78.7 (804)	82.3 (366)	
Marital status			<0.001				<0.001
Never married	66.3 (805)	55.8 (276)		88.6 (217)	48.6 (497)	82.7 (368)	
Ever married	33.7 (410)	44.2 (219)		11.4 (28)	51.4 (525)	17.3 (77)	
Employment status			<0.001				<0.001
Unemployed	39.8 (484)	37.0 (183)		49.0 (120)	34.6 (354)	43.6 (194)	
Student	12.1 (147)	4.7 (23)		16.3 (40)	7.1 (73)	12.8 (57)	
Self/salaried employment	17.4 (211)	18.8 (93)		16.7 (41)	18.9 (193)	15.7 (70)	
Laborer (agricultural/non-agricultural)	25.6 (311)	31.7 (157)		13.5 (33)	32.8 (335)	22.5 (100)	
Small business/trade	3.2 (39)	6.1 (30)		3.3 (8)	4.8 (49)	2.9 (13)	
Others^c^	1.9 (23)	1.8 (9)		1.2 (3)	1.8 (18)	2.3 (11)	
Age at first injection (years)			<0.001				< 0.001
< 18	5.7 (69)	7.7 (38)		4.9 (12)	5.2 (53)	9.4 (42)	
18–24	63.5 (772)	68.7 (340)		81.6 (200)	58.2 (595)	71.5 (318)	
25–29	20.1 (244)	19.0 (94)		11.8 (29)	23.3 (238)	16.2 (72)	
30+	10.7 (130)	4.7 (23)		1.6 (4)	13.3 (136)	2.9 (13)	
Frequency of injecting drugs^d^			0.002				0.059
Less frequent	45.1 (548)	37.0 (183)		38.4 (94)	45.0 (460)	39.8 (177)	
Frequent	54.9 (667)	63.0 (495)		61.6 (151)	55.0 (562)	60.2 (268)	

Column percentages are presented. *n* = total sample size for that category. ^a^Casual paid/unpaid sexual partner: women with whom IDUs had sex in the last 12 months in exchange for cash/drugs/kind and women other than main steady partner/spouse with whom IDUs had sex but did not pay. ^b^Inconsistent condom use: did not use a condom consistently in every sexual encounter. ^c^Others: includes other occupations such as unskilled labor, selling drugs. ^d^Frequent includes those who injected at least four times in the past week; less frequent includes those who injected less than four times in the past week.

Although IDUs reporting risky sexual behavior with casual paid/unpaid partners were less likely to have a regular sexual partner (42% vs 62%; adjusted OR = 0.41, 95% CI = 0.29–0.59) (Table 3), the odds of inconsistent condom use in sex with these partners were higher than among those who used condoms consistently with casual paid/unpaid partners (97% vs 66%; adjusted OR = 18.14, 95% CI = 6.82–48.21). Similarly, IDUs who did not have a casual paid/unpaid sexual partner in the past 12 months were significantly more likely to engage in unprotected sex with regular partners as compared to those who used condoms consistently in sex with casual paid/unpaid partners (92% vs 66%; adjusted OR = 2.87, 95% CI = 1.73–4.77). Additionally, IDUs who shared needles/syringes were more likely to engage in unprotected sex with regular partners than their counterparts (95% vs 87%; adjusted OR = 2.31, 95% CI = 1.30–4.09).

**Table 3:** Association between needle/syringe sharing, condom use with casual partners, and condom use with regular partners

	**Had a regular sexual partner**^**b**^		**Inconsistent condom use with regular sexual partner**^**a, b**^	
	**% ( *N *)**	**Adjusted OR (95% CI)**^**c**^	**% ( *N *)**	**Adjusted OR (95% CI)**^**c**^
Shared needles/syringes				
No	59.8 (1,215)	Ref	86.6 (726)	Ref
Yes	66.1 (495)	1.23 (0.94, 1.61)	94.8 (327)	2.31 (1.30, 4.09)^**^
Condom use with casual paid/unpaid sexual partner ^a, d^				
Consistent condom use	61.6 (245)	Ref	66.2 (151)	Ref
No casual paid/unpaid sexual partner	69.9 (1,022)	0.85 (0.61, 1.20)	91.9 (714)	2.87 (1.73, 4.77)^***^
Inconsistent condom use	41.9 (445)	0.41 (0.29, 0.59)^***^	97.4 (190)	18.14 (6.82, 48.21)^***^

Row percentages are presented. *OR* odds ratio, *CI* confidence interval. *p < 0.05, **p < 0.01, ***p < 0.001.

^a^Inconsistent condom use: did not use a condom consistently in every sexual encounter. ^b^Regular sexual partner: main/steady sexual partner (spouse or girlfriend) in the last 12 months. ^c^Adjusted for age, marital status, literacy, occupation, age at first injection, frequency of injecting drugs, and state; additionally shared needles/syringes and condom use with casual paid/unpaid sexual partner adjust for each other in this model. ^d^Casual paid/unpaid sexual partner: women with whom IDUs had sex in the last 12 months in exchange for cash/drugs/kind and women other than main steady partner/spouse with whom IDUs had sex but did not pay.

## Discussion

The findings of this study indicate that the majority of IDUs who have sex with casual female partners do not use condoms consistently in these relationships, findings similar to those reported in studies elsewhere in India [12,15]. The current study adds to this literature suggesting a significant association between IDUs' risky sexual practices with casual partners and non-condom use with regular sexual partners. Additionally, IDUs' who share injections/needles are more likely to engage in unprotected sex with their regular female partners, if they have such partners. These results provide evidence for the debate around the increase in HIV transmission to spouses from high-risk partners, for example, male IDUs in this study setting.

The finding that condom use practices are similar irrespective of sexual partner—IDUs who engage in unprotected sex with casual partners also report non-condom use with regular sexual partners—is of concern, as this could fuel the acquisition and transmission of HIV within these overlapping sexual networks. These findings are critical in light of recent estimates of new IDU epidemics emerging in selected states and districts of India [3] and indicate the need for HIV prevention interventions to go beyond addressing the risks associated with IDUs' needle-sharing practices to also focus on reducing the risk of sexual transmission of HIV in drug use settings. Special efforts are needed to reduce the vulnerability of IDUs' sexual partners to HIV, particularly their regular sexual partners, the majority of whom presumably are low-risk non-injecting women [11,18,19], who are currently not reached by HIV prevention programs due to the lack of programmatic learnings around reaching IDUs' regular sexual partners.

As seen in this study, one quarter of IDUs engage in unsafe injecting practices and the majority reported unprotected sex with both casual paid/unpaid as well as regular sexual partners. IDUs' unprotected sexual practices with regular partners may be due to the low-risk perception of HIV acquisition or transmission in such partnerships [19,25] or due to the fear of losing regular sexual partners if they disclose their risky injecting practices. Further research is needed to understand whether the regular female sexual partners of IDUs who engage in unsafe injecting practices have knowledge of HIV transmission and prevention. From the immediate programmatic perspective, peer educators need to counsel all IDUs on the risk of HIV transmission to regular sexual partners, if they do not adopt safe behaviors. A *post hoc* analysis suggests that about 3% of the total IDUs in the study districts not only shared injections and engaged in unprotected sex with casual paid/unpaid partners, but they also had sex with regular female partners. Every such respondent reported non-condom use in sex with regular female partners, indicating a high probability of HIV transmission within intimate partner relationships.

The results of this study also suggest that there are specific sub-groups of IDUs who engage in risky behaviors, including those in the younger age groups, not currently married, who inject frequently, and who had initiated injecting behaviors at a young age. Our findings on the characteristics of IDUs engaging in risky practices supports other published research studies, particularly regarding the early initiation into injecting drug use and frequency of injection increasing their HIV risk behaviors [17,18,26]. Efforts are needed to reach young, unmarried IDUs who are at high risk for HIV, through the use of young peer educators to encourage service utilization, and by organizing social activities that motivate young IDUs to take up risk reduction services [27].

Further, the results suggest the need to advance ongoing HIV prevention programs by identifying and reaching out to IDUs' regular sexual partners, to build awareness among such women about HIV risks and the ways to prevent infection. Available program resources for IDUs, such as the network of outreach workers, drop-in centers and voluntary counseling and testing centers, could be used to provide information and services to IDUs' regular sexual partners who are currently not covered by any HIV prevention programs. However, services may need to be appropriately designed to meet their needs. For example, drop-in centers for IDUs are perceived as providing services to high-risk males; as a result, IDUs' regular partners, who are mainly non-injecting women, may be reluctant to access these services for fear of being stigmatized; therefore, an effective strategy would be to set up female-friendly centers staffed entirely by women that provide a safe space for non-injecting women, which could also link women to other maternal health services [27]. Similarly, harm reduction programs for male IDUs currently use male outreach workers and male peer educators to provide services; however, to reach IDUs' sexual partners, female outreach workers and peer educators could be employed.

Although the study findings have important implications for HIV prevention programs and research, they should be considered in light of certain limitations. First, the odds ratios in this paper were derived from unweighted estimates because RDSAT software, which is generally used to analyze RDS data, cannot calculate bivariate or multivariate statistics; therefore, STATA was used for all analysis. Second, the findings of the study are based on self-reported data, which may be subject to social desirability bias, and as a result, socially unacceptable behaviors may have been underreported. However, the use of trained field staff may have increased study participants' comfort level at the time of interview and reduced under-reporting. Finally, the study findings cannot be generalized to all IDUs across the country as injecting drug practices in India vary across states. However, these limitations do not compromise the internal validity of the data.

In conclusion, our findings show that IDUs who engage in risky behaviors such as unsafe injecting practices and unprotected sex with casual partners are more likely to engage in unsafe sex with regular female partners, suggesting the urgent need to address HIV transmission within regular sexual partnerships. Ongoing risk reduction programs for IDUs need to expand their focus to include communication about condom use in all relationships in addition to addressing IDUs' unsafe injection practices in an effort to achieve the goal of zero new infections. An alternative and effective means to reach low-risk women, who are partners of individuals at high HIV risk, could be by providing HIV prevention services through available maternal and child health programs in India and elsewhere at the community level.

## Competing interests

The authors declare that they have no competing interests.

## Authors' contributions

All authors were responsible for study. RKM was responsible for the study conceptualization and led the writing of the paper. DG conducted the literature review and assisted in writing the paper. SR conducted the statistical analysis, assisted in interpreting the data and writing the paper. ML and LB assisted with interpretation of the results and drafting programmatic implications. CH was responsible for data collection and coordination of the study. NS co-led the conceptualization, supervised all aspects of writing the paper, and provided extensive comments on the manuscript. All the authors have read and approved the final manuscript.
